# Pregnancy outcomes in asylum seekers in the North of the Netherlands: a retrospective documentary analysis

**DOI:** 10.1186/s12884-020-02985-x

**Published:** 2020-05-25

**Authors:** A. E. H. Verschuuren, I. R. Postma, Z. M. Riksen, R. L. Nott, E. I. Feijen-de Jong, J. Stekelenburg

**Affiliations:** 1 Department of Obstetrics and Gynaecology and Department of Health Sciences, Global Health, University Medical Center Groningen/University of Groningen, Hanzeplein, 19713 GZ Groningen, the Netherlands; 2 Refaja ziekenhuis Stadskanaal, Boerhaavestraat, 19501 HE Stadskanaal, the Netherlands; 3 New Life, Sperwerlaan, 179561 BG Ter Apel, the Netherlands; 4 Department of General Practice & Elderly Medicine, University Medical Center Groningen, University of Groningen, Dirk Huizingastraat 3, 59713 GL Groningen, the Netherlands; 5 Department Obstetrics and Gynaecology, Medical center Leeuwarden, Henri Dunantweg 2, AD 8934 Leeuwarden, the Netherlands

**Keywords:** Asylum seekers, Pregnancy outcomes, Perinatal mortality, Vulnerable women, Substandard factors

## Abstract

**Abstract:**

**Background:**

With more than 20,000 asylum seekers arriving every year, healthcare for this population has become an important issue. Pregnant asylum seekers seem to be at risk of poor pregnancy outcomes. This study aimed to assess the difference in pregnancy outcomes between asylum seekers and the local Dutch population and to identify potential substandard factors of care.

**Methods:**

Using a retrospective study design we compared pregnancy outcomes of asylum-seeking and Dutch women who gave birth in a northern region of the Netherlands between January 2012 and December 2016. The following data were compared: perinatal mortality, maternal mortality, gestational age at delivery, preterm delivery, birth weight, small for gestational age children, APGAR score, intrauterine foetal death, mode of delivery and the need for pain medication. Cases of perinatal mortality in asylum seekers were reviewed for potential substandard factors.

**Results:**

A total of 344 Asylum-seeking women and 2323 Dutch women were included. Asylum seekers had a higher rate of perinatal mortality (3.2% vs. 0.6%, *p* = 0.000) including a higher rate of intrauterine foetal death (2.3% vs. 0.2%, p = 0.000), higher gestational age at birth (39 + 4 vs. 38 + 6 weeks, *p* = 0.000), labour was less often induced (36.9 vs. 43.8, *p* = 0.016), postnatal hospitalization was longer (2.24 vs. 1.72 days *p* = 0.006) and they received more opioid analgesics (27.3% vs. 22%, *p* = 0.029). Babies born from asylum-seeking women had lower birth weights (3265 vs. 3385 g, *p* = 0.000) and were more often small for gestational age (13.9% vs. 8.4%, *p* = 0.002). Multivariate analysis showed that the increased risk of perinatal mortality in asylum-seeking women was independent of parity, birth weight and gestational age at birth. Review of the perinatal mortality cases in asylum seekers revealed possible substandard factors, such as late initiation of antenatal care, missed appointments because of transportation problems, not recognising alarm symptoms, not knowing who to contact and transfer to other locations during pregnancy.

**Conclusion:**

Pregnant asylum seekers have an increased risk of adverse pregnancy outcomes. More research is needed to identify which specific risk factors are involved in poor perinatal outcomes in asylum seekers and to identify strategies to improve perinatal care for this group of vulnerable women.

## Background

With more than 20,000 asylum seekers arriving in the Netherlands per year, health care for this vulnerable population has become an important point of interest [1–3]. Asylum seekers are refugees whose request for sanctuary has not been processed yet by the country they seek refuge in [4]. Of all asylum seekers, 25% are women of reproductive age [5]. Research suggests that asylum-seeking women are disproportionately affected by health and social problems as compared to men, presumably because they are more vulnerable to physical assault and sexual harassment and they often feel their experiences and fears are not taken seriously [6].

A significant number of asylum-seeking women are or become pregnant during the time they seek refuge. Often, they arrive from countries with high rates of infectious disease, a poor health care system and have been persecuted, tortured or raped [7, 8]. During their flight circumstances are often primitive and dangerous. Once they arrive in the Netherlands, asylum seekers have little money, lack purpose in daily life and struggle with worries about the asylum procedure and the situation of family back home [7–9]. Research suggests that due to both the continuation of pre-existing health problems [10, 11] and the result of economic hardship and social deprivation once seeking residence [12, 13], asylum seekers have poorer physical and mental health compared to local populations [7, 14, 15]. Access to health care services is difficult for asylum seekers because of a lack of culturally appropriate information and a limited understanding of the Dutch health care system [9, 16].

Literature suggests that pregnancy outcomes are worse in asylum seekers, with higher perinatal and maternal mortality as compared to autochthonous populations [15, 17–20]. Studies in other countries show that refugee women have more complications such as low birth weight, low APGAR scores, preterm labour, anaemia, excessive bleeding during delivery and an increased incidence of Caesarean sections and admission of their child to the neonatal intensive care unit [11, 17, 20–24]. However, research on pregnancy outcomes on asylum seekers (i.e. those refugees whose request for sanctuary has not been processed yet) specifically is limited [25, 26]. In the Netherlands asylum-seeking women show a 10-fold increase in maternal mortality, twice as much perinatal mortality and an increased risk of maternal morbidity, including a higher prevalence of uterine rupture, eclampsia, major obstetric haemorrhage and intensive care unit admission during pregnancy [27–30]. Additional perinatal outcomes in asylum seekers in the Netherlands have not been assessed. This study aims to assess the current difference in perinatal outcomes between asylum seekers and the local Dutch population in an area in the North of the Netherlands with a high density of asylum seekers. Also, cases of perinatal mortality in asylum seekers were reviewed. This information may help to identify specific areas of interest in pregnancy care for asylum-seeking women and act as guidance for health care providers to meet the maternity care needs of this vulnerable population.

## Methods

### Study design

We performed a cross-sectional database study using one midwifery practice and hospital databases to assess maternal and perinatal outcomes of asylum seekers and the local Dutch population in the North of the Netherlands.

### Setting

In the Netherlands, asylum seekers live in asylum seekers centres during the processing of their request for sanctuary. In the North of the Netherlands, there are two major asylum seekers centres, Ter Apel and Musselkanaal. Ter Apel is the only central location of the Central Asylum Seeker Organization (COA; Centraal Orgaan Asielzoekers) in the Netherlands where asylum seekers who enter the country are accommodated at first instance. This resulted in a relatively high density of pregnant asylum-seeking women in the area.

### Maternity care in the Netherlands

In the Netherlands, low-risk pregnancies are followed up by primary care midwives and family doctors (for non-pregnancy related complaints) [31]. Primary care consists of monthly visits, a check every two weeks in the last phase of the pregnancy, and then every week [32]. Secondary and tertiary care could only be accessed with referral and includes hospital specialist care.

### Data collection

The data was retrieved from the primary and secondary care practices which provide the major part of pregnancy and delivery care to asylum-seeking women in Ter Apel and Musselkanaal. Databases were combined as described by Perined [33] to remove duplicate data of patients who were referred from primary to secondary care during pregnancy or delivery. Duplicate cases were identified by matching all cases on the mother’s date of birth, due date, duration of pregnancy and the country of origin of the mother.

### Study population

Asylum seekers who lived in Ter Apel or Musselkanaal and gave birth between January 2012 and December 2016 under the supervision of midwives from midwifery practice New Life or gynaecologists of the Refaja hospital were included in the study. Patients who were transferred to an asylum seeker centre elsewhere in the Netherlands before birth were excluded. The reference population consisted of the local Dutch population that gave birth under the supervision of the same care practices during the same time frame.

### Outcomes

Demographic factors were reported, including age, the number of adolescent pregnancies (19 years old or younger), country of origin, parity and uncertainty of due date. Parity was divided into 3 categories: nulliparous women, low multiparous women (1–3 previous deliveries) and grand multipara (> 3 previous deliveries). The outcome measures of this study were perinatal mortality (defined as death between 22 weeks of pregnancy and 7 days postpartum), maternal mortality, gestational age at delivery, preterm delivery (defined as delivery before 37 weeks of gestation), birth weight, small for gestational age children (SGA; defined as weight below the 10th percentile), APGAR score after 5 min, intrauterine foetal death (IUFD), start of labour (spontaneous, induction or primary caesarean section), mode of delivery (spontaneous, vacuum/forceps assisted delivery or secondary caesarean section) and pain medication (opioid and epidural analgesia). Also, the Adverse Outcome Index-5 (AOI-5) was calculated. The AOI-5 was designed to measure the magnitude of 5 adverse events that occurred during or around the delivery process [34]. It consists of perinatal mortality between a gestational age of 32 weeks and 7 days postpartum, neonatal intensive care unit (NICU) admission above 37 weeks, APGAR score lower than 7 after 5 min, postpartum haemorrhage and third- or fourth-degree perineal laceration. The AOI is defined as the number of women with one or more adverse outcomes during birth as a proportion of all deliveries. All cases of perinatal mortality in asylum seekers were reviewed, aiming to find potential substandard factors. Information from the different patient files was analysed using a structured approach. The checklist included the age of the mother, gestational age at birth, a case description and the results additional examinations like autopsy and amniocentesis. After a review of the patient files, the potential substandard factors were identified.

### Statistical analysis

All numerical values were tested for normality using Shapiro-Wilk’s Test. Since there were no normally distributed values, values were presented using the median and range. Categorical values were compared using Chi-square or Fishers Exact test. The Mann–Whitney U test was used to compare non-normally distributed and ordinal values. A logistic regression was performed to test for confounders for perinatal mortality. First, a univariate analysis was performed on possible confounding variables. All variables that showed a significant effect were included in a multivariate model. For the multivariate model a penalised likelihood logistic regression was used to reduce the chance of bias due to the low prevalence of perinatal mortality in our population. A value of *p < 0.05* was considered significant.

### Ethical considerations

As this is an anonymous retrospective database study there were no specific ethical issues to be considered. By law, this study does not fall under the Medical Research Involving Human Subjects Act in the Netherlands.

## Results

### Study population

Data of 2028 Dutch and 285 asylum-seeking women were included from the hospital database and 868 Dutch and 485 asylum-seeking women from the midwifery practice database. 216 (45%) pregnant women were transferred to an asylum-seeking centre elsewhere in the country before giving birth. After removing duplicates our study population included 2665 women: 344 asylum seekers and 2323 Dutch women.

### Demographic variables

Table 1 shows the demographic variables of both groups. Asylum seekers were younger (*p* = 0.000), had more adolescent pregnancies (*p* = 0.000) and there were more grand multipara women (*p* = 0.000) as compared to the control group. Most asylum seekers came from Syria (*n* = 75, 21.8%) and Eritrea (*n* = 65, 18.9%). Other countries were divided into categories based on geographical location.

**Table 1:** Demographic factors

Characteristics	Asylum seekers (*n* = 344)	Dutch population (*n* = 2323)	*p*-value
Age, years^a^			
Median	26	29	0.000
Range	14–42	15–45	
Adolescent pregnancy	30 (8.7)	48 (2.1)	0.000
Country of origin^b^			
Netherlands		2323 (100)	
Syria	75 (21.8)		
Eritrea	65 (18.9)		
Middle east	75 (21.8)		
Sub-Saharan Africa	50 (14.5)		
Eastern Europe and the former Republic of Yugoslavia	43 (12.5)		
Other	18 (5.2)		
Parity			0.000
Nulliparous	170 (49.4)	1141 (49.1)	
Low multiparous (1,2,3)	153 (44.5)	1147 (49.4)	
Grand multipara (≥4)	21 (6.1)	35 (1.5)	

Data are expressed as n (%) except where otherwise indicated

^a^Missing data: 1 from the Dutch population

^b^Missing data: 18 (5.2) from asylum seekers

### Pregnancy outcomes

Asylum seekers showed a higher rate of perinatal mortality (*p* = 0.000) including a higher rate of intrauterine foetal death (2.3% vs. 0.2%, p = 0.000), had a higher gestational age at delivery (*p* = 0.000), labour was less often induced (*p* = 0.016) and they more often received opioid analgesics (*p* = 0.029) as compared to Dutch women (Table 2). There was no significant difference in the frequency of epidural analgesia and APGAR scores after 5 min. There were no cases of maternal mortality. Babies born from asylum-seeking women had lower birth weights (*p* = 0.000), were more likely to be small for gestational age (*p* = 0.002) and there were more uncertain due dates in the asylum-seeking population (*p* = 0.000) (Table 2). After removal of cases with an uncertain due date, there was still no difference in prematurity between the two groups (*p* = 0.459). The adverse outcome index showed no difference between groups (*p* = 0.529) (Table 3). There were no significant differences in pregnancy outcomes between the different countries of origin except for parity (*p =* 0.001), gestational age at delivery (*p =* 0.034), the number of women with an uncertain due date (*p =* 0.036) and the use of an epidural (*p =* 0.005). Notable was that there were more grand multipara pregnancies in the Middle Eastern (10.7%) and the Eastern European (9.3%) groups and that the use of epidural anaesthesia during delivery was lower in the Eritrean group (see Appendix Table 6). Parity, gestational age at birth and birth weight showed a significant relation to perinatal mortality in univariate regression (Table 4) and were therefore included in a multivariate model (Table 5). After correction for these variables, asylum seekers were 7.2 times more likely to experience perinatal mortality as compared to Dutch women.

**Table 2:** Pregnancy outcomes

Indicator	Asylum seekers (*n* = 344)	Dutch population (*n* = 2323)	*p* value
Maternal mortality^a^	0 (0)	0 (0)	–
Perinatal mortality	11 (3.2)	14 (0.6)	0.000
Gestational age at delivery in days^b^			0.000
Median	277	272	
Range	166–302	112–296	
Uncertain due date^c^	114 (33.1)	52 (2.2)	0.000
Prematurity (< 37 weeks)	44 (12.8)	248 (10.7)	0.242
Birth weight^d^			0.000
Median	3265	3385	
Range	780–5050	920–5100	
SGA^e^	41 (13.9)	172 (8.4)	0.002
APGAR score after 5 min			0.054
Median (Mean)	10 (9.26)	10 (9.62)	
Range	0–10	0–10	
Postnatal hospitalization mother in days			0.006
Median (Mean)	1 (2.24)	1 (1.72)	
Range	0–20	0–27	
IUFD	8 (2.3)	4 (0.2)	0.000
Start of labour			
Spontaneous start	175 (50.9)	985 (42.4)	0.003
Inducing labour	127 (36.9)	1018 (43.8)	0.016
Primary caesarean section	42 (12.2)	320 (13.8)	0.429
Mode of delivery			
Spontaneous birth	188 (54.7)	1317 (56.7)	0.476
Vacuum or forceps assisted delivery	63 (18.3)	371 (16.0)	0.272
Caesarean section	93 (27.0)	635 (27.3)	0.907
Pain management			
Opioid analgesic	94 (27.3)	512 (22.0)	0.029
Epidural	79 (23.0)	438 (18.9)	0.072

Data are expressed as n (%) except where otherwise indicated

SGA, Small for gestational age; IUFD, Intrauterine foetal death

^a^Missing data: 58 from Asylum seekers and 296 from Dutch population

^b^Missing data: 1 from Dutch population

^c^Missing data: 4 from Dutch population

^d^Missing data: 2 from asylum seekers and 10 from the Dutch population

^e^Missing data: 49 from Asylum seekers and 275 from Dutch population

**Table 3:** Adverse Outcome Index-5

Indicator	Asylum seekers (*n* = 344)	Dutch population (*n* = 2323)	*p* value
Perinatal mortality (> 32 weeks and < 7 days postpartum) ^a^	2 (0.7)	2 (0.1)	0.078
APGAR score < 7 after 5 min^b^	12 (4.1)	41 (2.0)	0.023
NICU admission (> 37 weeks)^c^	5 (1.7)	16 (0.8)	0.171
Perineum Laceration (3rd or 4th degree) ^d^	4 (1.4)	46 (2.3)	0.346
Postpartum haemorrhage^e^	15 (5.2)	162 (7.9)	0.103
AOI-5 score^f^	33 (11.1)	257 (12.4)	0.529

*NICU* Neonatal intensive care unit, *AOI* Adverse outcome index

^a^Missing data: 57 from Asylum seekers and 295 from the Dutch population

^b^Missing data: 54 from Asylum seekers and 288 from the Dutch population

^c^Missing data: 54 from Asylum seekers and 289 from the Dutch population

^d^Missing data: 55 from Asylum seekers and 293 from the Dutch population

^e^ Missing data: 55 from Asylum seekers and 272 from the Dutch population

^f^Missing data: 48 from Asylum seekers and 256 from the Dutch population

**Table 4:** Univariate logistic regression predicting the likelihood of perinatal mortality

	B	S.E.	Wald	df	p-value	Exp(B) (Odds ratio)	95% C.I. for EXP(B)	
							Lower	Upper
Age	0.047	0.038	1.521	1	0.217	1.048	0.973	1.130
Parity	0.430	0.126	11.738	1	0.001	1.537	1.202	1.966
Asylum seeker	1.695	0.407	17.336	1	0.000	5.448	2.453	12.101
Gestational age at birth	−0.076	0.008	84.612	1	0.000	0.927	0.912	0.942
Birth weight	−0.003	0.000	88.012	1	0.000	0.997	0.996	0.998

**Table 5:** Multivariate penalised likelihood logistic regression predicting the likelihood of perinatal mortality

	﻿B	S.E.	Wald	df	p-value	Exp(B) (Odds ratio)	95% C.I. for EXP(B)	
							Lower	Upper
Constant	5.572	2.915	4.028	1	0.045	262.946	0.123	11.913
Asylum seeker	1.976	0.609	10.213	1	0.001	7.212	0.776	3.248
Birth weight	−0.002	0.001	7.599	1	0.006	0.998	−0.004	− 0.001
Gestational age at birth	−0.022	0.018	1.558	1	0.212	0.978	−0.061	0.012

Likelihood ratio test = 181.108 on 3 df, p = 0, n = 2654

Wald test = 88.535 on 3 df, p = 0

### Review of perinatal mortality cases

Of the eleven cases of perinatal mortality in asylum seekers, ten were intrauterine deaths and one child died within 24 h post-partum. The IUFD’s were diagnosed at the gestational age of 23 + 4, 23 + 6, 24, 25, 30 + 2, 34, 34 + 4, 34 + 6, 36 + 3 and 37 + 3 weeks, respectively. All women had their first antenatal care appointment after a gestational age of thirteen weeks, with an average of 22 + 3 weeks (*n* = 9, 2 unknown). Three women had no antenatal check-ups at all before an IUFD was discovered at respectively 33 + 2, 34 + 5 and 37 + 4 weeks. Another three women had one or more documented missed antenatal care appointments. Two women missed appointments because they were transferred to a different centre. None of the eleven women took the recommended dosage of folic acid during pregnancy; nine women did not take folic acid at all. Two women had a recorded history of mental health problems and in two cases there was substance abuse during pregnancy. In one of the cases, there was a multiple pregnancy with twin to twin transfusion syndrome. Intrauterine growth restriction was recorded in three cases and one woman developed preeclampsia. Further review of these cases revealed that in six cases there was a delay in seeking care when a woman experienced alarming symptoms: four women felt reduced foetal movement, for two and three days respectively, three weeks and a month before visiting a midwife. One of them did not know who to call during the weekend. One case of neonatal mortality within 24 h post-partum involved a woman who was losing green fluid with reduced foetal movement for three days, and an emergency caesarean section was performed because of signs of foetal distress. The child was born with APGAR scores of one after one, five and ten minutes and was anaemic. In six cases obduction was performed, which revealed two lightweight placentas showing maternal vascular malperfusion and one child had a trisomy 21.

## Discussion

This study aimed to assess the difference in maternal and perinatal outcomes between asylum seekers and the local Dutch population in the North of the Netherlands and identify potential substandard factors in the care for asylum seekers. In this study perinatal mortality was higher in asylum seekers, birth weight and APGAR scores were lower and postnatal hospitalization was longer compared to Dutch women. Labour in asylum seekers was less often induced, opioid analgesics were administered more often and there were more adolescent pregnancies. There was no difference in preterm birth rate and the mode of delivery, nor in the adverse outcome index. No cases of maternal mortality were recorded in this study. Overall the findings of our study were in line with previous research [15, 18–21, 23, 25, 30–32]. We found that, even after correcting for confounders, perinatal mortality was higher in asylum seekers. Review of these cases revealed possible substandard factors causing a delay in the first two phases of the Three Delays Model (deciding to seek care and reaching the healthcare facility [35]), consisting of late initiation of antenatal care, missed appointments because of problems with transportation, not recognizing alarm symptoms, not knowing who to contact and transfer during pregnancy. Our findings re-enforce those from previous studies and also identify additional delays in the third step of the model (receiving adequate care), such as a language barrier, fear of mistreatment, shame and non-availability of a female doctor [11, 13, 15, 23, 36–38]. All these factors may contribute to the limited use of antenatal care in asylum-seeking women [13, 18, 20, 39–41]. Poor attendance to antenatal care has been associated with poor pregnancy outcomes [42, 43]. Previous studies showed that a lack of antenatal care results in lower folic acid intake [44], which has been described to increase the risk of low birth weight [45]. This may have played a role in our population as we indeed found lower birth weight and a higher prevalence of SGA children in asylum seekers. Our study was not powered to detect a difference in maternal mortality since the incidence of maternal mortality in the Netherlands is 7 deaths per 100,000 live births [46]. Previous studies suggest that maternal mortality is higher among asylum seekers [13, 15, 27, 28].

We found that the use of opioid analgesics was higher in asylum-seeking women as compared to the Dutch group. A potential reason for this may be because coaching these women can be a bigger challenge for a caregiver due to a language barrier and cultural differences. However, there was no difference in the use of epidural anaesthesia. In our study, the rate of labour induction was higher in Dutch women. Previous research showed conflicting results about the difference in labour induction between groups [39, 47]. The option of labour induction after 41 weeks of gestation is discussed with patients in the Netherlands. It is possible that asylum-seeking women might not know of the possibility, due to less antenatal care visits and a language barrier. In our study, postnatal hospital stay was significantly longer in asylum-seeking mothers as opposed to other studies [20, 48]. A lack of facilities and social support at home could contribute to this.

This study did not observe differences in the incidence of preterm birth, low APGAR scores, adverse outcome index, NICU admission, perineum laceration, postpartum haemorrhage, mode of delivery and the rate of epidural analgesia. Previous studies showed similar results, except for a higher rate of epidural analgesia use during labour in local populations and lower APGAR scores in asylum seekers [17, 20, 23, 39, 47, 49].

Finally, ours and other studies showed that asylum-seeking women were on average younger and had a higher parity rate [17, 20, 23, 39, 47, 48]. The higher parity rate in asylum seekers could be attributed to cultural differences and little control over family planning decisions, including access to contraceptives [18]. Other studies showed that grand multipara women had a higher incidence of maternal morbidity and therefore poorer perinatal outcomes [50] however in our study, parity and age showed no relation to perinatal mortality in multivariate analysis. An uncertain estimated date of delivery was more common in asylum seekers because of a lack of ultrasounds in early pregnancy. Therefore, the rate of premature children and SGA in this study could be underestimated. We found no significant differences in pregnancy outcomes between the different countries of origin in the asylum-seeking group.

### Strengths and limitations

This was the first study comparing a wide range of pregnancy outcomes in asylum seekers and Dutch women. This study included asylum seekers from different countries of origin, while previous studies often included asylum seekers from one specific background. Our sample size (*n* = 2665) was large compared to previous studies whose sample sizes were all smaller than 1500 women with only two previous studies exceeding 1000 participants.

A language barrier plays a role with most pregnant asylum seekers, however, because of the retrospective character of the study there was not sufficient information to which extent this language barrier played a role and if and how often official translator services were used. Maternal mortality was also not assessed in this study. Our group was too small to assess the difference in maternal mortality and maternal morbidity between groups. For this study, we only included data from one hospital and one midwife practice. However, these facilities have vast experience in caring for asylum seeking women since the asylum-seeking centre in Ter Apel is the largest centre in the Netherlands. Also, the control group consisted of women from the northern Netherlands which was a region where a relatively large proportion of the population had a low socioeconomic status. Therefore, the control group might not have been representative of the general Dutch population. Differences in outcomes between the general Dutch population and asylum seekers may be even larger.

### Implications for care providers

This study highlights the importance of improving care for pregnant asylum seekers. Extra attention should be paid to asylum-seeking women during pregnancy by health care providers with the ultimate goal to achieve equity in health. Our study identifies possible substandard factors of the current care system which could facilitate the development of effective health care interventions. Alternative forms of antenatal care for asylum-seeking women targeting the identified substandard factors should be developed. In the North of the Netherlands, we are currently working with a group antenatal care program specifically for asylum-seeking women.

In this study 45% of the asylum seekers were transferred to another centre during pregnancy causing discontinuation of antenatal care. Transfer between asylum-seeking centres during pregnancy should be minimized to reduce suboptimal care for an already vulnerable population.

### Further research

To provide more data about perinatal outcomes in asylum seekers, larger prospective multicentre studies should be conducted. Comparing the difference in perinatal outcome between different countries of origin might give insight in which women within the asylum-seeking population are extra vulnerable. Since antenatal care use is limited in asylum seekers, alternative forms of antenatal care and its effect on pregnancy outcomes should be studied. We are currently studying whether group antenatal care as compared to standard antenatal care in the Netherlands improves pregnancy outcomes and satisfaction with care in asylum seekers. Also, psychosocial factors and the incidence of mental health problems in asylum seekers should be studied.

## Conclusion

Perinatal outcomes in asylum seekers appear to be worse compared to Dutch women. Extra attention should be paid to pregnant asylum seekers to make sure quality maternity care is provided. This study highlights that reducing disparities in pregnancy outcomes between asylum seekers and Dutch women should be an important public health goal in the Netherlands. Further large-scale research should be conducted to improve antenatal care for pregnant asylum seekers and to identify specific risk factors for poor perinatal outcomes in asylum seekers. Alternative forms of antenatal care and its effect on pregnancy outcomes in asylum seekers need to be studied.

## Data Availability

The datasets used and analysed during the current study are available from the corresponding author on reasonable request.

## Appendix

**Table 6:** differences in pregnancy outcomes between different origins

Characteristics	Eritrea (***n*** = 65)	Syria (***n*** = 75)	Middle east (***n*** = 75)	Sub-Saharan Africa (***n*** = 50)	Eastern Europe (***n*** = 43)	Other (***n*** = 18)	***p***-value
**Age, years**							0.592
Median	25	26	25	29	28	26	
Range	17–42	14–41	17–41	19–41	18–38	18–40	
**Adolescent pregnancy**	6 (9.2)	7 (9.3)	6 (8.0)	2 (4.0)	4 (9.3)	2 (11.1)	0.895
**Parity**							0.001
Nulliparous	44 (67.7)	31 (41.3)	42 (56.0)	26 (52.0)	11 (25.6)	8 (44.4)	
Low multiparous (1,2,3)	20 (30.8)	41(54.7)	25 (33.3)	22 (44.0)	28 (65.1)	9 (50.0)	
Grand multipara (≥4)	1 (1.5)	3 (4.0)	8 (10.7)	2 (4.0)	4 (9.3)	1 (5.6)	
**Maternal mortality**	0 (0)	0 (0)	0 (0)	0 (0)	0 (0)	0 (0)	–
**Perinatal mortality**	1 (1.5)	4 (5.3)	3 (4.0)	2 (4.0)	0 (0)	0 (0)	0.535
**Gestational age at delivery in days**							0.034
Median	280	275	275	279	279	270	
Range	203–302	175–295	167–294	166–302	229–294	251–294	
**Uncertain due date**	29 (44.6)	20 (26.7)	25 (33.3)	17 (34.0)	7 (16.3)	8 (44.4)	0.036
**Prematurity (< 37 weeks)**	6 (9.2)	9 (12.0)	5 (6.7)	7 (14.0)	6 (14.0)	4 (22.2)	0.452
**Birth weight**^a^							0.122
Median	3343	3200	3300	3140	3320	3123	
Range	1665–4320	780–4245	1780–4395	1415–4360	1662–5050	2305–4685	
**SGA**^b^	8 (12.3)	9 (12.0)	6 (8.0)	7 (14.0)	6 (14.0)	3 (16.7)	0.892
**APGAR score after 5 min**							0.491
Median (Mean)	10 (9.31)	10 (9.24)	10 (9.29)	10 (9.00)	10 (9.58)	10 (9.61)	
Range	0–10	0–10	0–10	0–10	6–10	4–10	
**Postnatal hospitalization mother in days**^c^							0.168
Median (Mean)	1.5 (2.17)	1 (2.51)	1 (1.88)	2 (2.21)	1 (2.00)	2 (3.06)	
Range	0–10	0–20	0–10	0–10	0–12	1–8	
**IUFD**	1 (1.5)	3 (4.0)	2 (2.7)	1 (2.0)	0 (0)	0 (0)	0.734
**Start of labour**							0.534
Spontaneous start	30 (46.2)	32 (42.7)	45 (60.0)	25 (50.0)	23 (53.5)	8 (44.4)	
Inducing labour	29 (44.6)	31 (41.3)	24 (32.0)	18 (36.0)	16 (37.2)	6 (33.3)	
Primary caesarean section	6 (9.2)	12 (16.0)	6 (8.0)	7 (14.0)	4 (9.3)	4 (22.2)	
**Mode of delivery**							0.529
Spontaneous birth	30 (46.2)	41 (54.7)	47 (62.7)	25 (50.0)	29 (67.4)	9 (50.0)	
Vacuum or forceps assisted delivery	16 (24.6)	12 (16.0)	13 (17.3)	9 (18.0)	5 (11.6)	4 (22.2)	
Caesarean section	19 (29.2)	22 (29.3)	15 (20.0)	16 (32.0)	9 (20.9)	5 (27.8)	
**Pain management**							
Opioid analgesic	22 (33.8)	14 (18.7)	23 (30.7)	15 (30.0)	8 (18.6)	7 (38.9)	0.174
Epidural	6 (9.2)	26 (34.7)	23 (30.7)	10 (20.0)	9 (20.9)	2 (11.1)	0.005

Data are expressed as n (%) except where otherwise indicated

*SGA* Small for gestational age, *IUFD*, Intrauterine foetal death

Missing data: 18 asylum seeking women had no recorded country of origin so were not included in this table

^a^Missing data: 1 Middle Eastern and 1 Sub Saharan Africa

^b^Missing data: 10 Eritrea, 11 Syria, 9 Middle Eastern, 5 Sub Saharan Africa, 5 Eastern Europe and 2 other

^c^Missing data: 1 Eritrea, 1 Syria, 6 Middle Eastern, 3 Sub Saharan Africa, 3 Eastern Europe and 2 other

## Abbreviations

AOI: Adverse outcome index
IUFD: Intrauterine foetal death
NICU: Neonatal intensive care unit
SGA: Small for gestational age
